# Measuring health-related quality of life of older people with frailty receiving acute care: feasibility and psychometric performance of the EuroQol EQ-5D

**DOI:** 10.1186/s12873-023-00909-4

**Published:** 2023-11-19

**Authors:** James D. van Oppen, Simon P. Conroy, Timothy J. Coats, Nicola J. Mackintosh, Jose M. Valderas

**Affiliations:** 1 College of Life Sciences, George Davies Centre, University of Leicester, Leicester, UK; 2 Emergency and Specialist Medicine, University Hospitals of Leicester NHS Trust, Leicester, UK; 3 MRC Unit for Lifelong Health and Ageing, University College London, London, UK; 4 Department of Family Medicine, National University Health System, Singapore, Singapore

**Keywords:** Frailty, Geriatric emergency medicine, Patient-reported outcome, Quality of life

## Abstract

**Background:**

Although outcome goals for acute healthcare among older people living with frailty often include Health-Related Quality of Life (HRQoL) and other patient-reported outcome measures (PROMs), current quality metrics usually focus on waiting times and survival. Lay and patient review have identified the EuroQol EQ-5D as a candidate measure for this setting. This research appraised the EQ-5D for feasibility, psychometric performance, and respondents’ outcomes in the acute frailty setting.

**Methods:**

People aged 65 + with Clinical Frailty Scale (CFS) 5–8 were recruited from eight UK hospitals’ emergency care and acute admissions settings. They completed the five-level EQ-5D and the EQ-VAS. Feasibility was assessed with completion times and completeness. For reliability, response distributions and internal consistency were analysed. Finally, EQ-Index values were compared with demographic characteristics and service outcomes for construct validity.

**Results:**

The 232 participants were aged 65–102. 38% responded in emergency departments and 62% in admissions wards. Median completion time was 12 (IQR, 11) minutes. 98% responses were complete. EQ-5D had acceptable response distribution (SD 1.1–1.3) and internal consistency (Cronbach’s alpha 0.69). EQ-VAS demonstrated a midpoint response pattern. Median EQ-Index was 0.574 (IQR, 0.410) and was related positively with increasing age (*p* = 0.010) and negatively with CFS (*p* < 0.001). Participants with higher CFS had more frequent problems with mobility, self-care, and usual activities.

**Conclusions:**

Administration of the EQ-5D was feasible in these emergency and acute frailty care settings. EQ-5D had acceptable properties, while EQ-VAS appeared problematic. Participants with more severe frailty had also poorer HRQoL.

## Introduction

Acute care encompasses emergency departments, urgent care, and acute admissions ward settings. Traditionally this operationalises ‘single-problem’ rapid-flowing pathways and is increasingly recognised to have limitations in serving older people living with frailty, who have multiple problems and require a more holistic approach [1]. Geriatric emergency medicine is emerging as a multidisciplinary subspecialty with its basis in person-centred care, wherein interventions are selected and tailored for individuals’ situations and perspectives [2–4]. Health-Related Quality of Life (HRQoL) is often the primary outcome goal of acute care episodes for older people living with frailty [5, 6]. This population group have poorer outcomes from acute care, but these have typically been qualified with system metrics including survival times, waiting times, and readmission rates rather than with HRQoL measures [7, 8].

Incorporating HRQoL measurement into healthcare metrics could facilitate more meaningful evaluation of outcomes for older people living with frailty. This could be achieved using Patient-Reported Outcome Measures [9]. Systematic reviews of PROMs have reported lower quality of life among people living with frailty in community settings [10, 11]. However, there is little knowledge regarding their HRQoL in the acute care setting, explained in part by the lack of any validated PROM for unwell older people living with frailty [9, 12]. A comprehensive PROM (or a combination) for this population would consider themes of autonomy and function [13]. In a systematic review and lay evaluation of potentially suitable PROMs for those themes of function, the EuroQol 5 Dimension (EQ-5D) presented an acceptable compromise between detail and brevity [14].

The EQ-5D has been administered in acute care research and audit programmes with specific groups of older people, notably those with trauma [15, 16]. Its feasibility has been demonstrated in community, outpatient, general medical, and focussed surgical settings [17]. However, it has yet to be evaluated for feasibility or psychometric properties during use by older people with frailty receiving acute healthcare – a setting uniquely epitomised by urgency and uncertainty.

EQ-5D invites respondents to rate the severity of problems experienced in the five dimensions mobility, self care, usual activities, pain / discomfort, and anxiety / depression [18]. People living with frailty are vulnerable to poor outcomes from health crises. Reduced independent function and heightened concern and discomfort would typically be expected among people receiving care following sudden change in situation due to acute illness or injury. These symptoms and their subsequent trajectory could be measured using the EQ-5D.

This evaluation of EQ-5D in emergency departments and acute admissions ward settings took place within a larger research programme aiming to improve understanding of the lived experience of older people receiving acute healthcare. This paper reports on objectives to (1) assess the feasibility of collecting EQ-5D from acutely unwell older people living with frailty, (2) assess the psychometric performance of the EQ-5D in this setting, and (3) describe HRQoL and its relationship with frailty in this population.

## Methods

This was an analysis of PROM, demographic, and overview clinical service data. The study was nested within a programme developing a novel PROM for older people living with frailty receiving acute care (the PROM-OPAC), in which the EQ-5D was administered as a comparator instrument during two phases of administration.

Eligible participants were older people (aged 65 +) who were receiving care in hospital within seventy-two hours of unscheduled attendance. Precise locations included emergency departments, observation areas, and acute assessment wards. They were living with frailty, defined as having Clinical Frailty Scale (CFS) scores 5 (mild frailty) to 8 (very severe frailty) [19]. People with terminal illness who otherwise did not have severe frailty (CFS = 9) were not recruited. The recruiting sites routinely collected the CFS for people aged 65 + receiving acute care. Routine CFS measurements by nursing or medical staff were used by research practitioners to identify eligible individuals.

Participants were approached and recruited by research practitioners using convenience sampling. They provided written consent, given either themselves or by their consultee in the case of having capacity to complete the measure but not to consent to research. This included, for instance, people living with cognitive impairment who were able to express their perspectives and perceptions but who could not fully process or retain the research information or complete a consent form. The study protocol was reviewed and approved by an NHS Research Ethics Committee.

### Data collection

Data were collected at eight UK hospitals. These were located in London and the South East (4), East Midlands (3), and the North West (1) of England. Participants’ age, CFS, gender, and ethnicity (using UK Office for National Statistics groups) were recorded. All participants completed PROMs using pen and paper, with or without the assistance of a scribe: the five-level EQ-5D-5L (which evaluates problems with mobility, self-care, usual activities, pain and discomfort, and anxiety and depression), and the EQ-VAS (a 1–100 scale of the user’s health that day). The administered instruments used English language. All responses were from participants and proxies were not used. Time to completion (from commencing instrument responses) was recorded.

The degree of assistance required by the first half of participants was rated by the research practitioner as none, minimal (demonstration only), moderate (reading aloud or scribing), or substantial (explaining options or prompting for responses). Meanwhile for the second half of participants, the single-item Self-Rated Health (SRH) was also collected, and clinical service data were recorded (presenting problem, location in hospital, time from attendance to participation, and destinational outcomes from the ED and after thirty days) [20].

### Analyses

EQ-Index values (preference-weighted utilities for economic evaluation anchored at 1 (best health) and 0 (a state as bad as being dead)) were calculated from complete responses using a crosswalk value set [21]. Analyses were performed using R software with the packages *eq5d*, *ggplot2*, and *psych* [22].

Feasibility was first assessed. Times to completion were examined for normality, summarised, and compared with age and CFS using correlation and Kruskal–Wallis tests. Missing data were analysed for frequency and pattern.

Next, reliability was determined. EQ-5D data were analysed for response distributions (response level proportions and standard deviations) and internal consistency (question inter-relatedness using Cronbach’s alpha statistic) [23].

Finally, criterion and construct validity were examined. EQ-Index was assessed for convergence with EQ-VAS (all respondents) and SRH (second half of participants). EQ-Index was compared with age, CFS, gender, and ethnicity using correlation, Kruskal–Wallis, and chi-squared tests. The proportions of respondents with severe or extreme problems in each dimension were aggregated by CFS levels. EQ-Index and dimension-level proportions of severe problems for the second half of participants were tested for association with waiting time at participation, presenting problem (dichotomised as illness or injury), disposition destination from the ED, and clinical outcome after thirty days. Associations were hypothesised with lower ED EQ-Index and EQ-VAS for people with longer waits, injury presentations, subsequent hospital admissions, and subsequent reattendance or death within thirty days.

## Results

Two hundred and thirty-two participants were recruited: 38% responded in emergency departments and 62% in acute admissions ward settings. Participants’ age ranged from 65 to 102 years, and they were living with mild to very severe frailty. More participants had mild to moderate (CFS 5, 31%, CFS 6, 50%)) than severe or very severe frailty (CFS 7, 18%; CFS 8, 1%). This approximated the expected distribution of CFS among older people receiving emergency care [24]. The sample recruited more females (62%), and nearly all participants reported white ethnicity (Table 1).

**Table 1:** Characteristics of recruited participants

	Cohort *N* = 232
Female, n (%)	143 (62%)
Age /years, median (range)	85 (65–102)
Ethnicity group other than white, n (%)	4 (2%)
Clinical Frailty Scale	
*5 (living with mild frailty)*	*71 (31%)*
*6 (living with moderate frailty)*	*117 (50%)*
*7 (living with severe frailty)*	*41 (18%)*
*8 (living with very severe frailty)*	*3 (1%)*
Location at time of administration (for *N* = 118 participants in second recruitment stage)	
*Emergency department*	*45 (38%)*
*Acute admissions wards*	*73 (62%)*

### Feasibility

Median time for participants to complete the PROMs was 12 (IQR, 11) minutes. Completion time was weakly correlated positively with increasing age (Spearman *rho* = 0.15, *p* = 0.022) and was not associated with CFS (Kruskal–Wallis *p* = 0.358). There were minimal missing data, with 98% EQ-5D responses complete. The item ‘usual activities’ had the most frequent missing responses (1%). 102 of 128 (80%) first half participants used at least minimal assistance – 94 being supported with reading or scribing and 8 requiring explanations or prompts.

### Psychometric properties

Ceiling effects were observed with the ‘self care’ and ‘anxiety / depression’ questions, respectively with 35% and 42% participants reporting no problems (Table 2). Response distribution remained satisfactory (standard deviation > 0.95) for all EQ-5D items. There was a midpoint response pattern with EQ-VAS, where 23% participants responded ‘50’. EQ-5D internal consistency was acceptable (α = 0.69).

**Table 2:** EQ-5D item response distribution (standard deviation and proportions at each level) and internal consistency (Cronbach’s raw alpha statistic overall and with each item dropped)

**Item**	**SD**	**Response level**						α = 0.69
		**1**	**2**	**3**	**4**	**5**	**Missing**	**α if dropped**
Mobility	1.1	0.03	0.21	0.32	0.28	0.16	0	0.62
Self Care	1.3	0.35	0.20	0.27	0.08	0.10	0	0.60
Usual Activities	1.4	0.16	0.23	0.23	0.19	0.19	0.01	0.63
Pain / Discomfort	1.2	0.23	0.17	0.34	0.20	0.06	0	0.67
Anxiety / Depression	1.1	0.42	0.25	0.23	0.06	0.03	0	0.68

Responses were scored 1 (no problems) to 5 (extreme problems) for each dimension

EQ-Index converged with both the EQ-VAS (Spearman *rho* = 0.277, *p* < 0.001) and the SRH (Kruskal–Wallis *p* = 0.053).

### Health-related quality of life

#### Relationships with participants’ characteristics

The median EQ-Index in this cohort was 0.574 (IQR, 0.410) and this was similar for participants recruited across the eight sites (chi-squared *p* = 0.170). 11 participants (5%) had EQ-Index lower than 0, corresponding to states worse than being dead. EQ-Index increased weakly with age (Spearman *rho* = 0.170, *p* = 0.010), and decreased with more severe frailty (Kruskal–Wallis *p* < 0.001). There was no association between EQ-Index and gender (chi-squared *p* = 0.244) and the recruited ethnicity distribution was insufficient for association testing.

**Fig. 1:**
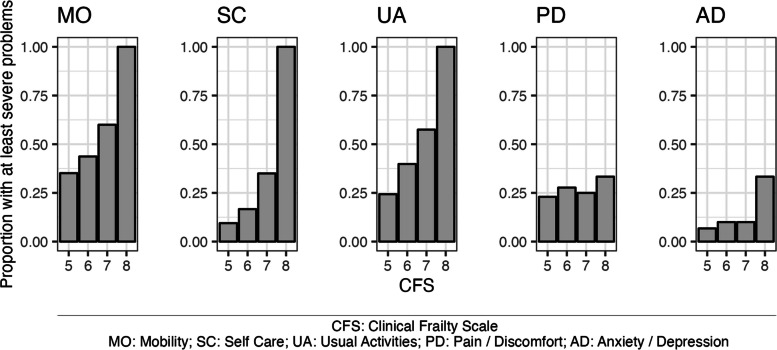
Dimension-level proportions of severe or extreme EQ-5D problems with Clinical Frailty Scale (CFS) level

With increasing CFS, participants more frequently reported severe or extreme problems with mobility (Kruskal–Wallis *p* = 0.010), self care (*p* < 0.001), and usual activities (*p* = 0.001). All participants living with very severe frailty (CFS 8) reported at least severe problems in these dimensions, although it should be noted that these were a very small proportion of the cohort. No association was apparent between frailty and having severe problems with pain and discomfort (*p* = 0.955) or anxiety and depression (*p* = 0.327) (Fig. 1).

#### Associations with clinical outcomes

Participants who participated at later points during the seventy-two hour acute care phase tended to have higher EQ-Index (Spearman *rho* = 0.229, *p* = 0.029). There was no significant difference in EQ-Index between respondents attending with illnesses or injuries (chi-squared *p* = 0.534). Similarly, responses did not significantly differ between groups admitted or discharged from the ED (chi-squared *p* = 0.334), or with different thirty-day clinical outcomes (chi-squared *p* = 0.240).

## Discussion

This study adds to a limited evidence base considering HRQoL and its measurement in acutely unwell older people living with frailty. The objective was not to advocate for EQ-5D as a perfectly suited outcome measure for older people living with frailty; indeed, previous work has shown it to consider meaningful themes of function but not of autonomy [14]. However, collection of the EQ-5D from older people living with frailty receiving acute care appeared feasible. Measurements appeared reliable and the responses appeared valid against objective observation of frailty.

The high proportion of fully completed five-level EQ-5D responses observed here (98%) exceeded that (90%) reported in a recent systematic review of use by older people, where the three-level variant was most frequently administered [17]. All participants were accompanied by a research practitioner who offered assistance as a scribe. With 80% participants using this assistance, this intervention likely improved the rates of full completion and allowed people with common accessibility barriers to respond. Further work is required to evaluate completion rates among acute care respondents when support is not offered. The practical implications on representation and data quality of providing scribe support in routine PROMs programmes will also require consideration.

The adequate psychometric properties of response distribution and internal consistency for EQ-5D were consistent with previous reports in studies of older people living with frailty [25, 26]. However, the midpoint response pattern with EQ-VAS appears novel in this acute care application and is problematic. This may represent difficulty interpreting instructions or appreciating one’s health trajectory in the context of illness and uncertainty, or may indeed be an effect of feeling rushed by research practitioner presence.

Poorer mean (SD) EQ-Index values have been reported by people living with versus without frailty in the UK (0.71 (0.21) vs 0.92 (0.10)) and Vietnam (0.58 (0.20) vs 0.70 (0.18)) [27, 28]. The present sample of older people living with frailty and receiving acute care had still poorer EQ-Index (median 0.58), with 5% having negative values corresponding to situations worse than being dead. Having poorer HRQoL has been associated with increased mortality [29]. Similarly, the predictive performance for mortality of the Clinical Frailty Scale in ED has been widely reported [24, 30]. HRQoL and the CFS appear linked as objective and subjective manifestations of the frailty construct, wherein lived biographies are disrupted by functional deficits and uncertainty [31, 32]. While HRQoL has been observed to improve during geriatric ward admissions, further evaluation of person-reported outcomes from acute care is warranted [33, 34].

### Limitations

This research recruited only those older people who were living with frailty. As such, the design did not allow direct HRQoL comparison with people who were younger or not living with frailty, and further evaluation is therefore required during and following acute illness. Furthermore, we did not study for the potential roles of educational attainment or socioeconomic deprivation. It is possible that these users of acute healthcare may have differed from the general population, with those having altered baseline physiology and function being more likely to experience crises. Baseline HRQoL may therefore also differ in people who subsequently require acute care, and this would require a large-scale cohort study to evaluate.

While people with impaired capacity were recruited with consultee consent, the study did not examine for the effect of cognitive impairment on response rate, completeness, or reported outcomes. This was a limitation of the study being conducted within a PROM development programme. While people living with cognitive impairment did participate, only individuals who could express their perspectives by responding or indicating responses to the items were recruited. The study design did not extend to scoring quantifying the severity of cognitive impairment, but we can assume that people with severe limitations were not represented here. Around one third of older people using the emergency department might be expected to have a dementia or delirium, and further work is required to determine accessibility and feasibility of PROMs participation with this cohort [35]. Other PROMs have used proxy completion to represent people living with dementia [36]. Acknowledging potential issues with precision, this approach may further widen the access to participation and is a topic for further study.

Calculation of EQ-Index used a value set produced from UK population-level data. These are not necessarily comparable to other populations. Furthermore, the precision of these values, which fall at the extreme-low end of the population range, should be considered cautiously in comparative studies. Validation and application of a value set focussed on an older population may be more meaningful.

All participants were recruited and accompanied by a research practitioner, which introduced potential selection and acquiescence biases. Research practitioners may have preferentially approached individuals who they thought would be better able to complete the instruments. People without fluent English, for instance, may have been excluded from participation and therefore further research using consecutive recruitment is indicated. While the administered PROMs did not directly invite participants to report on their healthcare experience, this design may still have prompted participants to report better outcomes in the presence of a professional.

## Conclusions

Older people living with frailty were able to complete the EQ-5D and EQ-VAS while receiving acute care in emergency departments and admissions wards, with excellent data completeness. EQ-5D had adequate psychometric properties, while a midpoint response pattern was observed with EQ-VAS. This sample of older people living with frailty receiving acute healthcare had poor HRQoL, which was negatively associated with having increasingly severe frailty.

## Data Availability

The datasets generated and analysed during this study are not publicly available due to the ethics permissions received but are available from the corresponding author on reasonable request.
